# Synthesis of chiral polymorph A-enriched zeolite Beta with an extremely concentrated fluoride route

**DOI:** 10.1038/srep11521

**Published:** 2015-06-22

**Authors:** Mingquan Tong, Daliang Zhang, Weibin Fan, Jun Xu, Liangkui Zhu, Wen Guo, Wenfu Yan, Jihong Yu, Shilun Qiu, Jianguo Wang, Feng Deng, Ruren Xu

**Affiliations:** 1 State Key Laboratory of Inorganic Synthesis and Preparative Chemistry, Department of Chemistry, Jilin University, Changchun, 130012, China; 2 State Key Laboratory of Coal Conversion, Institute of Coal Chemistry, Chinese Academy of Sciences, Taiyuan, 030001, China; 3 State Key Laboratory of Magnetic Resonance and Atomic and Molecular Physics, Wuhan Institute of Physics and Mathematics, Chinese Academy of Sciences, Wuhan, 430071, China

## Abstract

Chiral zeolitic materials with intrinsically chiral frameworks are highly desired because they can combine both shape selectivity and enantioselectivity. In the field of zeolite, the synthesis of chiral polymorph A of zeolite Beta or chiral polymorph A-enriched zeolite Beta is one of the biggest challenges. We demonstrate here a generalized extremely concentrated fluoride route for the synthesis of chiral polymorph A-enriched zeolite Beta in the presence of five achiral organic structure-directing agents. The polymorph A-enriched Ti-Beta shows a higher enantioselectivity for the asymmetric epoxidation of alkenes than the normal Ti-Beta.

Zeolites and related microporous crystalline materials, which have periodic one-to-three-dimensional frameworks and well-defined pore structures, have attracted significant interest due to their wide range of applications in catalysis, ion exchange, separation, and adsorption^1^,^2^. Chiral zeolitic materials with intrinsically chiral frameworks^3^,^4^ are of particular interest because they can combine both shape selectivity and enantioselectivity, which are desirable for enantioselective catalysis and separation^5^ and fundamental aspects in chirality^6^. Therefore, significant effort has been devoted to the synthesis of chiral zeolitic materials during past decades. To date, several zeolite frameworks with intrinsic chirality, including *BEA^7^,^8^, CZP^9^, GOO^10^, -ITV^11^, JRY^12^, LTJ^13^, OSO^14^, SFS^15^, and STW^16^,^17^,^18^, have been synthesized in the presence of chiral or achiral organic structure-directing agents (OSDAs). Zeolite Beta was first synthesized by Mobil in 1967 with tetraethylammonium hydroxide (TEAOH) as an OSDA^19^. Its structure was independently determined by Newsam *et al.*^7^ and by Higgins *et al.*^8^ in 1988. According to the structural analysis, zeolite Beta is an intergrowth of two distinct but closely related polymorphs, i.e.polymorphs A and B, in a ratio of ca. 44:56. In polymorph A, the stacking of the centrosymmetric tertiary building layer occurs in either a left-handed or a right-handed fashion. The cages are arranged in a helical fashion around a fourfold screw axis forming a helical channel along the c-axis of the tetragonal unit cell in either a right- or left-handed manner. In polymorph B, the stacking occurs as a recurrent alternation in right- and left-handed fashion forming an achiral structure. Based on the same building layers, four more polymorphs of zeolite Beta family were proposed, including polymorph C^7^, C_H_^8^, D^20^,^21^, and E^20^,^21^. Polymorph C possesses no translations of the centrosymmetric tertiary building layers in either the *a* or *b* direction. The stacking of two such layers makes double four-membered rings (D4R) cages. Polymorph D, and E also contain the D4R cages, which are not found in either polymorph A, B, or C_H_. To date, pure polymorph C^22^, nearly pure polymorph B^23^, and the intergrowth zeolite Beta of polymorph B and C_H_^21^ have been synthesized. Intergrown zeolite Beta of D and E has been recently reported, which gave two intense X-ray diffraction peaks at 2θ = 7.0 and 9.7° 24. However, chiral polymorph A has not been synthesized yet. Given the valuable potential applications of chiral polymorph A, much effort has been devoted to the synthesis of pure polymorph A or polymorph A-enriched zeolite Beta. Unfortunately, the success is limited. The first effort was reported by Davis *et al.* in 1992^25^. The authors suggested a strategy for synthesizing one enantiomorph of polymorph A. By using an unspecified chiral OSDA, the authors synthesized a zeolite Beta enriched in polymorph A, which was determined from a comparison of the experimental XRD pattern of the resulting zeolite Beta with the simulated pattern of the pure polymorph A. This sample of zeolite Beta was capable of performing enantioselective adsorption and catalysis, yielding an enantiomeric excess of 5%. In recent years, some groups have claimed the synthesis of polymorph A-enriched zeolite Beta in the presence of chiral or achiral OSDAs^26^,^27^,^28^. However, the profiles of the experimental XRD patterns they have reported are very similar to that of the normal zeolite Beta.

In this work, we present a generalized route for the synthesis of chiral polymorph A-enriched zeolite Beta in extremely concentrated fluoride medium. With this route, chiral polymorph A-enriched zeolite Beta was obtained in the presence of five OSDAs, i.e., tetraethylammo-nium hydroxide (TEAOH), N,N-dimethyl-2,6-cis-dimethylpiperdinium hydroxide (DMPOH), dimethyldiisopropylammonium hydroxide (DMDPOH), N,N,N-trimethylcyclohexanaminium hydroxide (TMCHOH), and N-ethyl-N,N-dimethylcyclohexanaminium hydroxide (EDMCHOH) as shown in Scheme S1 (Supporting information). The polymorph A-enriched Ti-Beta shows enantioselectivity for the asymmetric epoxidation of alkenes as compared to the normal Ti-Beta.

## Results and Discussion

### Physicochemical properties of the polymorph A-enriched zeolite Betas

**Figure 1:**
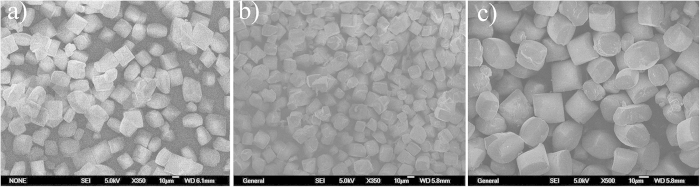
SEM images of Beta-TEAOH (a), Beta-DMPOH (b), and Beta-DMDPOH (c). The scale bar in the SEM images represents 10 μm.

The SEM images of Beta-TEAOH (polymorph A-enriched zeolite Beta directed by TEAOH), Beta-DMPOH, and Beta-DMDPOH are shown in Fig. 1, which suggest that the bulk samples are free of impurities. The high crystallinity of the bulk samples was confirmed by their ^29^Si MAS NMR spectra (Figure S1, Supporting Information). For comparison, the ^29^Si MAS NMR spectrum of normal zeolite Beta^28^ (directed by TEAOH) is also provided in Figure S1. No signals higher than −105 ppm were found, which indicates the absence of connecting defects due to Q_3_ sites^29^. The signals in the range of −109 ~ −116 ppm are assigned to the crystallographically different Q_4_ sites^28^.

### The polymorph structures of the polymorph A-enriched zeolite Betas

#### The XRD analysis of the polymorph A-enriched zeolite Betas

**Figure 2:**
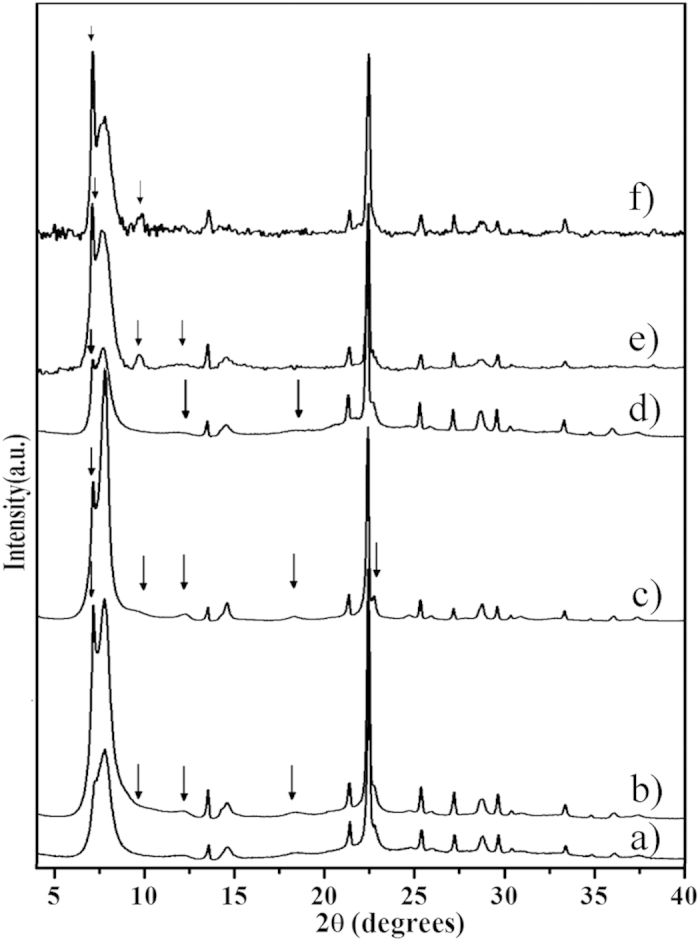
Powder XRD patterns of the calcined normal zeolite Beta (a), Beta-TEAOH (b), Beta-DMPOH (c), Beta-DMDPOH (d), Beta-TMCHOH (e), and Beta-EDMCHOH (f). The arrows show the characteristic peaks related to the chiral polymorph A of zeolite Beta.

The powder XRD patterns of the as-synthesized polymorph A-enriched Beta and normal zeolite Beta are shown in Fig. 2. The profile of the patterns of the polymorph A-enriched zeolite Beta is similar to that of the highly crystallized normal zeolite Beta except for the presence of some characteristic peaks related to the chiral polymorph A, as marked with arrows. The absence of the intense peak at 2θ = 9.7° suggests that the samples do not include the polymorphs of D and E^24^. The broadening of the peaks in the low-angle region is due to the stacking faults in the intergrowth of polymorphs A and B. The first broad peak at low angle is regarded as the overlap of four peaks (Cu-Kα) at 2θ = 6.98° and 7.74°, which are assigned to the (100) and (101) planes of polymorph A respectively, and 2θ = 7.34° and 8.31°, which are assigned to the (110) and (−111) planes of polymorph B respectively. Compared to polymorph B, polymorph A generates four characteristic peaks (2θ = 6.98° (100), 9.67° (102), 12.23° (103), and 18.18° (105); see Figure S2). In contrast, polymorph C shows two characteristic peaks at 2θ = 6.90° (100) and 9.68° (101), which are very close to those of polymorph A (Figure S2). Therefore, the possibility of the existence of polymorph C in the investigated bulk samples cannot be excluded solely on the results of the powder XRD analysis. Nevertheless, according to the results obtained from Figure S2 and the XRD pattern of SSZ-63^21^, the existence of polymorph C_H_ can be excluded. Polymorph C contains D4R cages, which exhibit high affinity to fluoride ions.

#### The ^19^F MAS NMR analysis of the polymorph A-enriched zeolite Betas

**Figure 3:**
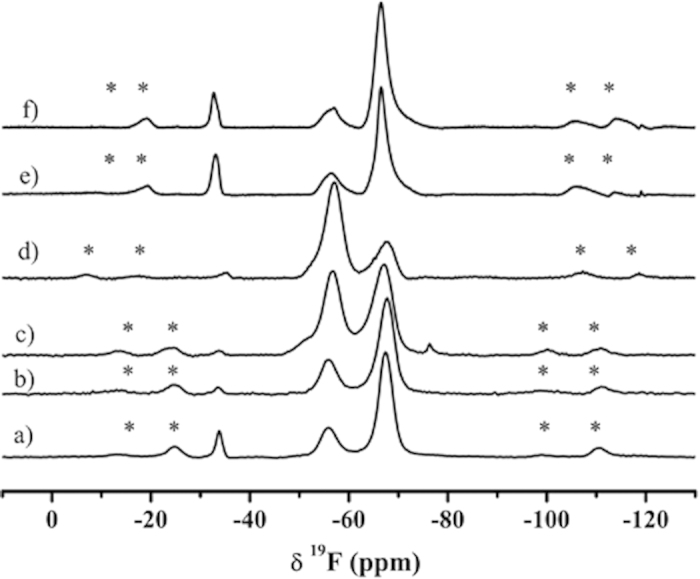
^19^F MAS NMR spectra of normal zeolite Beta (a), Beta-TEAOH (b), Beta-DMPOH (c), Beta-DMDPOH (d), Beta-TMCHOH (e), and Beta-EDMCHOH (f). The asterisks indicate spinning side bands.

Fig. 3 shows the ^19^F MAS NMR spectra of normal zeolite Beta and polymorph A-enriched Beta-TEAOH, Beta-DMPOH, Beta-DMDPOH, Beta-TMCHOH, and Beta-EDMCHOH. Three signals at −36, −57 and −68 ppm are observed in all of these ^19^F MAS NMR spectra. The first signal is due to the fluoride ion occluded in the D4R cage^20^,^22^, whereas the latter two signals are attributed to the fluoride ions occluded in the [4^3^5^4^] cages of zeolite Beta^20^. Because neither polymorph A nor polymorph B contains D4R cages, the detected D4R cages in the normal zeolite Beta and Beta-TEAOH are most likely associated with the existence of the at least two adjacent layers stacked with the manner in polymorph C. In addition, the ^19^F signal at −36 ppm in the spectra of Beta-TEAOH (Fig. 3b), Beta-DMPOH (Fig. 3c), and Beta-DMDPOH (Fig. 3d) is much less intense than the corresponding signal in the spectrum of normal Beta (Fig. 3a); while the intensity of this signal in the spectra of Beta-TMCHOH (Fig. 3e) and Beta-EDMCHOH (Fig. 3f) is comparable to that in the spectrum of normal Beta (Fig. 3a). This suggests that the content of polymorph C in polymorph A-enriched Beta-TEAOH, Beta-DMPOH, and Beta-DMDPOH is far lower than that in normal Beta, and the content of polymorph C in polymorph A-enriched Beta-TMCHOH and Beta-EDMCHOH is comparable to that in normal Beta.

#### The DIFFaX simulation analysis of the polymorph A-enriched zeolite Betas

The ratio of polymorph A to polymorph B in zeolite Beta is usually estimated by comparing the experimental XRD pattern with the simulated XRD patterns with different A/B ratios. The simulation is commonly performed using the DIFFaX computer program developed by Treacy *et al.*^7^ Thus, the XRD patterns of zeolite Beta with different A/B ratios are simulated (Figure S3) by this program. Fig. 2 and Figure S2 shows that the low-angle peaks are broadened by the fault stacking of different polymorphs. The low-angle region is widely accepted as the fingerprint area of zeolite beta. By comparing the shapes of the broadened low-angle peaks, the polymorph compositions could be determined. According to the simulated XRD patterns, the proportion of polymorph A in the Beta-TEAOH, Beta-DMPOH, and Beta-DMDPOH was approximately 65%, 65%, and 60%, respectively. The appearance of the characteristic peak of 2θ = 9.67° in the XRD patterns of Beta-TMCHOH and Beta-EDMCHOH suggests that they have a higher proportion of polymorph A (~70%).

#### The HRTEM analysis of the polymorph A-enriched zeolite Beta

**Figure 4:**
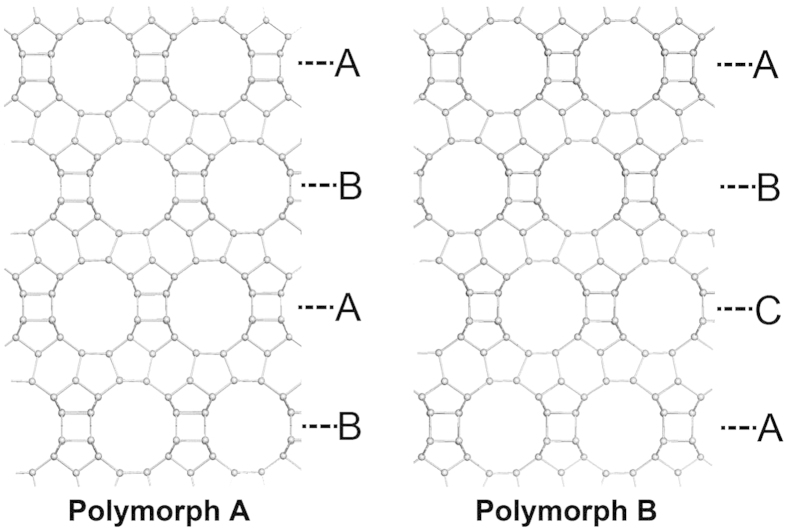
Structures of polymorph A and polymorph B of zeolite Beta that show the different stacking sequences of the 12-MR ring channels as ABAB… and ABCABC… types, respectively.

X-ray diffraction patterns provide information concerning the long-range ordering of a crystal, but in the case of zeolite Beta, investigation of its local structure may be more important because it may reveal the distribution of polymorphs A and B. Therefore, high-resolution transmission electron microscopy (HRTEM) was used to analyze the local structure of polymorph A-enriched zeolite Beta. As has been well documented^7^, the structure of Beta zeolite is built from a centrosymmetric tertiary building layer that consists of 12-membered ring (MR) structures. It contains channels that intersect in three directions with 12-MR channels. When viewed along the <100> directions, ABAB… type and ABCABC… type stacking sequences of the 12-MR channels are expected for polymorph A and polymorph B of zeolite Beta respectively (Fig. 4).

**Figure 5:**
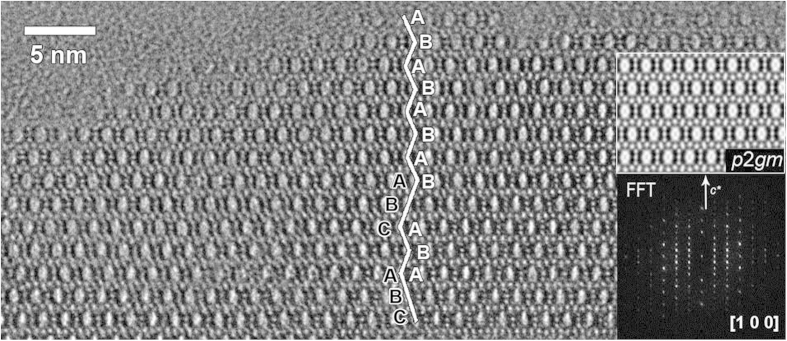
HRTEM image of a thin edge of the Beta-TEAOH crystal shows an enriched area of polymorph A type stacking. The stacking sequences of the 12-MR channels are marked in the image. The insert is a FFT pattern generated from the thin area of the image, in which the dominance of polymorph A clearly shows.

HRTEM images were taken from the thin boundaries of Beta-TEAOH crystals along the <100> directions. One of the representative HRTEM images is shown in Fig. 5, in which the stacking of 12-MR channels is marked. Although a few stacking layers that follow the ABCABC… type stacking sequence was observed, a Fast Fourier Transform (FFT) pattern generated from the HRTEM image clearly shows the dominance of polymorph A. The lattice-averaged projection derived from the pure ABAB… stacking region shows *p2gm* projection symmetry and is consistent with the space group *P*4_1_22 (*P*4_3_22). Well-resolved 4-, 5-, and 6-MR structures were observed from the lattice-averaged projection images.

**Figure 6:**
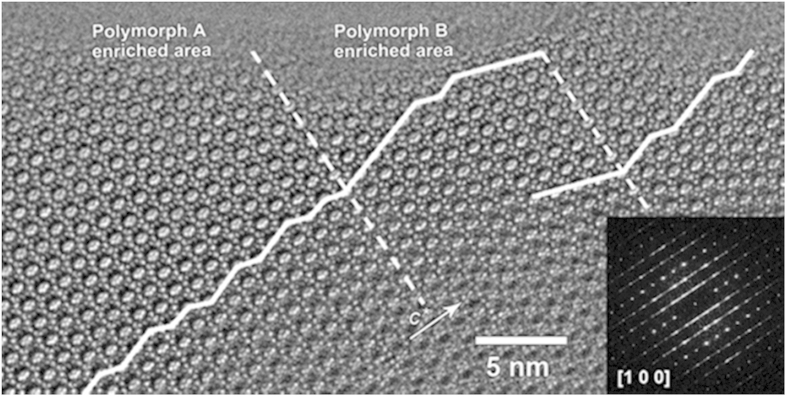
HRTEM image taken along the <100> direction of a broken crystal piece of Beta-TEAOH. Guidelines are added in the image to mark the stacking sequences of the 12-MR channels. The dashed line represents the partition between the polymorph A-enriched and polymorph B-enriched areas. The insert is a FFT pattern generated from the thin area of the image, in which the reflections are elongated along the c* direction.

To investigate the intergrowth features inside the bulk crystal, HRTEM images were also taken from broken crystal pieces of Beta-TEAOH. Both ABAB… type and ABCABC… type stacking sequences were observed in these images (Fig. 6), as marked using white lines. The alternations of areas dominated by polymorphs A and B were observed, which strongly indicates the intergrowths inside the bulk crystals. As expected, diffused reflection lines were found from the FFT pattern generated from the HRTEM image in Fig. 6. However, no polymorph C-type zeolite Beta, which should exhibit an AAA… stacking sequence in the <100> projections, was observed in the TEM studies. Statistical counting from several HRTEM images taken along the <100> directions gave an averaged ratio of approximately 3:2 of stacking sequences of polymorph A to that of polymorph B, being coincided well with that determined from the powder XRD patterns. The intergrowth features and the polymorph A enrichment were also observed.

**Figure 7:**
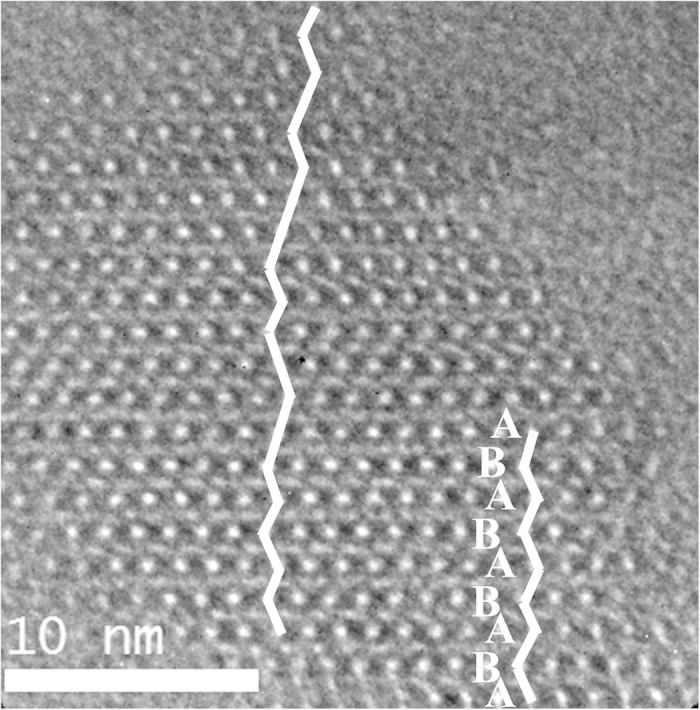
HRTEM image taken from a broken crystal piece of a Beta-DMPOH crystal along the [100] direction. Guidelines are added to mark the stacking sequences of the 12-MR channels.The ABAB… stacking sequences show the polymorph A domain.

An enrichment in polymorph A means a larger overall proportion of A domains, which could be achieved by either enlarging A domains or increasing the probability of small A domains. However, in numerous HRTEM researches, the polymorph A domains were not larger than 2 unit cells for the synthesized zeolite Beta^7^,^30^,^31^,^32^ and only a 3 unit cells of polymorph A domain was observed in the tschernichite^33^, the nature counterpart of zeolite Beta. In our synthesized polymorph A-enriched zeolite Beta, the regions with up to 4 unit cells of polymorph A were observed in the HRTEM images of Beta-DMPOH (Fig. 7) and Beta-TEAOH (Fig. 5), suggesting the increase in the probability of polymorph A did translate in larger A domains.

### Asymmetric epoxidation of 1-phenyl-1-cyclohexene and β-methylstyrene over polymorph A-enriched Ti-Beta

Polymorph A of zeolite Beta contains two enantiomorphs, which belong to the space groups of *P*4_1_22 and *P*4_3_22. If these two enantiomorphs exist with the same possibility, the sample would not exhibit enantioselectivity in chiral catalysis or separation. Therefore, the determination of whether the polymorph A-enriched zeolite Beta contains reasonable enantiomeric excess is important and necessary. If the polymorph A-enriched zeolite Beta is enantioenriched to a reasonable degree, enantioselectivity would be observed in the chiral catalysis reaction. Therefore, we prepared the Ti-Beta-TEAOH and used it to catalyze the asymmetric epoxidation of β-methylstyrene and 1-phenyl-1-cyclohexene. For comparison, the normal Ti-Beta with the same Ti/Si ratio in the initial synthesis mixture was also prepared. The polymorph A percentage of the Ti-Beta-TEAOH was estimated with the DIFFaX simulated method (Figure S4).

The catalytic results obtained over the polymorph A-enriched Ti-Beta-TEAOH and normal Ti-Beta for asymmetric epoxidation of 1-phenyl-1-cyclohexene and β-methylstyrene are summarized in Table 1 and Table S1 respectively. The normal Ti-Beta samples with about 50% polymorph A percentage give negligible ee values for the SS and RR epoxides (0.12% and 0.5%) in the epoxidation of β-methylstyrene, while Ti-Beta-TEAOH with about 65% polymorph A gave an ee value of 4.76% or 5.67% (Table S1). In the asymmetric epoxidation of 1-phenyl-1-cyclohexene (Table 1), the ee values for the cis-epoxides obtained on the polymorph A-enriched Ti–Beta-TEAOH are also much higher than that of the normal Ti-Beta, and the highest ee value exceeds 10% (11.40%), despite that no quantitative relation was observed between the ee value and the polymorph A content. Figure S5 shows the diffuse reflectance (DR) UV-vis spectra of all the Ti-Beta catalysts. It is clear that the Ti coordination states of different samples are significantly different, consequently resulting in the samples with great difference in the catalytic performance. A125-1 and A125-2 with large amounts of octahedral Ti species gave a conversion of 1-phenyl-1-cyclohexene of 2.29% and 7.50%, respectively (Table 1), being as low as about 1/9 and 1/2.74 of that of N125-1. This is due to not only their lower Ti contents but also lower TONs. It is worth pointing out that A125-1 and A125-2 prepared with the synthesis gels having similar amounts of Ti exhibit considerably different enantioselectivity. This probably arises from the presence of different amounts of extraframework octahedral Ti species in A125-2 than in A125-1 (Figure S5). The octahedral Ti species does not show enantioselectivity. In addition, the enantioselectivity of the polymorph A-enriched zeolite beta not only is related to the polymorph A content, but also directly depends on the relative amount of two enantiomorphs with space groups of *P*4_1_22 or *P*4_3_22. In the synthesized normal zeolite beta, the polymorph A domains are usually smaller than 2 unit cells. Hence, the two enantiomorphs would exist with the same probability, resulting in a negligible enantioselectivity. In the synthesized polymorph A-enriched zeolite beta, although the polymorph A domains were enlarged, the enantioselectivity are still dependent on the relative amount of the two enantiomorphs. Therefore, a significant difference in the enantioselectivity of A125-1 and A125-2 is also caused by the relative amounts of the two enantiomorphs, which cannot be determined yet for the moment.

### The effects for the crystallization of polymorph A-enriched zeolite Betas

TEAOH is a default OSDA for the normal zeolite Beta. Under the synthesis conditions presented here, however, TEAOH can direct polymorph A-enriched zeolite Beta. Compared to the initial mixture for the synthesis of normal zeolite Beta, the water content in the initial mixture for the crystallization of polymorph A-enriched zeolite Beta was extremely low. The mixture was dried in an 80 °C oven before it was loaded into the autoclave. After drying at 80 °C for a certain time, the color of the mixture changed to light-grey, which suggests a partial decomposition of TEAOH via Hofmann degradation. To quantitatively investigate the water content in the initial mixture and how much TEAOH was decomposed, the eight different initial mixtures were subjected to thermogravimetric analysis (TGA). The TGA curves for these initial mixtures are provided in Figure S6. The results of the TGA analysis of these eight initial mixtures are summarized in Table S2. To estimate the ratio of TEAOH and possible triethylamine (TEA) in the initial mixture and the evolution of the TEAOH during the crystallization process, the initial mixture and the highly crystallized Beta-TEAOH were analyzed by ^13^C MAS NMR, and the highly crystallized Beta-TEAOH was also analyzed by TGA. The corresponding ^13^C MAS NMR spectra are shown in Figures S7 and S8, and the TGA curve is shown in Figure S9. The ^13^C MAS NMR spectrum in Figure S7 clearly shows that the initial mixture contains TEAOH and TEA. However, the result in Figure S8 undoubtedly shows that the organic species in highly crystallized Beta-TEAOH is TEA^+^, which suggests that the TEA is not included in Beta-TEAOH. According to Figure S7 we can approximately determine that the molar ratio of TEAOH/TEA is approximately 2/3 (0.61). Based on the TGA data in Table S2, the typical batch composition of the initial mixture can be determined to be SiO_2_:0.19TEAOH:0.31TEA: 0.29H_2_O. Considering the water introduced by the HF aqueous solution in the following step, the molar composition of the final initial mixture for the crystallization of Beta-TEAOH is SiO_2_:0.19TEAOH:0.31TEA:1.12H_2_O:0.5HF. The TGA analysis on the highly crystalline Beta-TEAOH (Figure S9) suggests that each unit cell (Si_64_O_128_) contains 5.67 TEAF, which gives a formula of SiO_2_·0.09TEAF. This number is consistent with previously published results^29^. Compared to the synthesis conditions used for the normal zeolite Beta, the concentration of the reactants used in this route was extremely high. This difference may play an important role in affecting the preference of the stacking sequence of the centrosymmetric tertiary building layer.

It is well known that TEAOH is a default OSDA of normal zeolite Beta. Compared to the initial mixture for the synthesis of normal zeolite Beta, the water content in the initial mixture for the crystallization of polymorph A-enriched zeolite Beta was extremely low. This difference may play an important role in affecting the preference of the stacking sequence of the centrosymmetric tertiary building layer. This is supported by the facts that DMPOH, DMDPOH, TMCHOH, and EDMCHOH can also direct the polymorph A-enriched zeolite Beta under present conditions, and that an increase in the water content in the synthesis gel led to formation of normal zeolite Beta not matter what OSDA was used.

In summary, we developed a generalized extremely concentrated fluoride-route for the synthesis of chiral polymorph A-enriched zeolite Beta with five OSDAs. With this route, chiral polymorph A-enriched Ti-Beta was synthesized. This material exhibited a reasonably higher enantioselectivity than normal Ti-Beta in the asymmetric epoxidation of alkenes. The extremely high concentrated condition introduced in this route for enriching the chiral polymorph of zeolite Beta may work in the synthesis of one polymorph of other intergrowth zeolites.

## Methods

### Synthesis of OSDAs

In addition to the TEAOH, other four OSDAs were used in the synthesis of polymorph A-enriched zeolite Beta. Substitution reactions were used to synthesize the OSDAs. Take the synthesis of DMPOH as an example. Typically, 1.0 mol cis-2,6-dimethylpiperidine (Alfa Aesar) reacted with 2.2 mol of methyl iodide (Beijing Chemical Cop.) and 1.0 mol of KHCO_3_ in methanol at ambient temperature for 4–5 days. After the reaction was completed, the methanol solvent was removed under reduced pressure. The solid mixture was extracted for three times with chloroform. The chloroform solution was then dried with sodium sulfate for 2–3 hours. After filtration, the chloroform was removed under reduced pressure, and the product was recrystallized in a minimum amount of hot methanol. The solid product of DMPI was then exchanged with OH-type ion-exchange resin to obtain aqueous quaternary ammonium hydroxide solutions, DMPOH. The synthesis of DMDPOH, TMCHOH and EDMCHOH are similar to that of DMPOH, except that diisopropylamine (Beijing Chemical Cop.), methylcyclohexylamine (aladdin-reagent) and ethylcyclohexylamine (aladdin-reagent) were used for DMDPOH, TMCHOH and EDMCHOH, respectively.

### Synthesis of the pure silica form of polymorph A-enriched zeolite Beta

The procedures for the synthesis of polymorph A-enriched zeolite Beta with DMPOH, DMDPOH, TMCHOH, and EDMCHOH were identical to those used for the synthesis of sample with TEAOH, which is as an example described in the following. Typically, tetraethylorthosilicate (TEOS, 99 wt%) was added to aqueous TEAOH solution (35 wt%) with TEOS : TEAOH molar ratio of 1.0: 0.5. The mixture was then stirred at ambient temperature for 4 hours for hydrolysis of TEOS. This is followed by stirring under an infrared lamp to evaporate generated ethanol and most of the water. The thick gel was subsequently placed in an 80 °C oven for 3–6 days until the H_2_O/SiO_2_ ratio reached approximately 0.3 with the colour of the solid becoming light-grey. The solid was ground into a powder, and then, hydrofluoric acid (HF, 40 wt%, Beijing Chemical Cop.), 0.5 times of the TEOS molar, was drop-wise added under continuous manually stirring conditions (Caution: HF is known to be highly corrosive and toxic. Though no problems have been encountered during this experiment, great caution should be paid.). Finally, the uniform mixture was transferred into an autoclave and heated at 150 °C for 4–7 days. The solid product was washed thoroughly with water and dried at 100 °C overnight. The template was removed by calcination in static air at 560 °C for 6 h with a heating rate of 5 °C min^−1^.

### Synthesis of the pure silica form normal zeolite Beta

First, tetraethylorthosilicate (TEOS, 99 wt%) was added to aqueous TEAOH solution (35 wt%). After hydrolysis of TEOS about 4 hours, hydrofluoric acid (HF, 40 wt%, Beijing Chemical Cop.) was drop-wise added under stirring conditions. Finally, the uniform mixture with the composition SiO_2_:0.5TEAF:7.5H_2_O was transferred into an autoclave and heated at 140 °C for 6–10 days. The solid product was washed thoroughly with water and dried at 100 °C overnight. The template was removed by calcination in static air at 560 °C for 6 h with a heating rate of 5 °C min^−1^.

### Synthesis of polymorph A-enriched and normal Ti-Beta in the presence of TEAOH

Polymorph A-enriched and normal Ti-Beta were synthesized according to the methods used for synthesis of the polymorph A-enriched zeolite Beta and the normal zeolite Beta except that the aqueous H_2_O_2_ solution (35 wt%) and tetraisopropyltitanate with molar ratio about 25:1 were introduced orderly after the TEOS being added.

### Characterization Techniques

Powder X-ray diffraction (XRD) patterns were recorded on a Rigaku D/MAX-2500 diffractometer with a CuKα radiation source (λ = 0.15418 nm) operated at 50 kV and 200 mA. The morphology of the samples was observed on a scanning electron microscope (SEM) (JEOL JSM-6700F). The HRTEM images were collected on a JEM-3010 transmission electron microscope (spherical aberration constant Cs = 0.6 mm) operated at 300 kV and were recorded by a bottom-mounted CCD camera (Gatan MultiScan Camera Model 794). A double tilt holder (tilt ranges x: ±25° and y: ±23.5°) was used for tilting the crystal to the desired orientation. The crystallographic image processing was performed using the program CRISP^34^. ^29^Si, ^13^C, and ^19^F MAS NMR spectra were measured on a Varian Infinityplus-300 spectrometer with resonance frequencies of 299.8, 282.0, 75.4, and 59.6 MHz for ^1^H, ^19^F, ^13^C, and ^29^Si, respectively. A Chemagnetics 4 mm double-resonance MAS probe was used to acquire the ^19^F NMR spectra, and a 7.5 mm triple-resonance MAS probe was used for the ^29^Si and ^13^C experiments. ^29^Si single-pulse experiments were conducted with a 4.5 μs pulse width (π/2) and a 100 s recycle delay. ^19^F MAS spectra were acquired using a DEPTH pulse sequence with a π/2 pulse of 3.8 μs and a recycle delay of 80 s with a spinning rate of 16 kHz. ^13^C MAS NMR single-pulse experiments were performed using a 4.3 μs pulse width (π/2) and a 12 s recycle delay. The chemical shifts were referenced to methylsilane (TMS) for ^29^Si, trifluoroacetic acid for ^19^F, and adamantane for ^13^C.

### Catalysis experiments

The asymmetric epoxidation of alkenes over the Ti-Beta-TEAOH and normal Ti-Beta was carried out under the following conditions: 0.2 g catalyst, 10 mL acetonitrile, substrate/H_2_O_2_ (35% in aqueous solution) = 1, 70 °C, 2 h. The products were identified by a Shimadzu GCMS-QP 2010 Ultra instrument equipped with a Rtx-5MS column and/or comparing with standard (2S,3S) and (2R,3R)-epoxide. Enantioselectivities were determined by chiral GC (Shimadzu, GC-2010 Plus) equipped with a CP-Chirasil-Dex CB column.

## Additional Information

**How to cite this article**: Tong, M. *et al.* Synthesis of chiral polymorph A-enriched zeolite Beta with an extremely concentrated fluoride route. *Sci. Rep.* **5**, 11521; doi: 10.1038/srep11521 (2015).

## Supplementary Material

Supplementary Information

## Figures and Tables

**Table 1:** Catalytic results obtained over the polymorph A-enriched Ti-Beta-TEAOH and normal Ti-Beta for asymmetric epoxidation of 1-phenyl-1-cyclohexene.

Cat. No.^a^	Si/Ti^b^	Poly A(%)^c^	Conv. (%)	TON	Selec.^d^				ee_epo_%^e^
					1	2	3	4	
A125-1	126	67	2.29	3.25	73.80	2.70	3.22	15.75	−8.75
A125-2	–	66	7.50	–	68.17	2.07	1.89	22.22	4.77
A60-1	58	64	22.66	6.53	53.64	6.79	5.96	30.05	6.53
A60-2	–	62	20.41	–	66.29	2.24	1.78	25.72	11.40
N125-1	94	52	20.53	7.52	59.74	3.72	3.64	28.53	1.04
N60-1	42	50	15.68	5.25	49.01	6.56	6.75	33.18	−1.41

^a^“A” and “N” represent polymorph A-enriched Ti–Beta-TEAOH and normal zeolite Beta, respectively; “125” and “60” represent the Si/Ti ratios in the synthesis gels. The final digit (like in A125-1 and A125-2) denotes a different sample from a different batch.

^b^The Si/Ti ratios of the bulk samples were determined by ICP analysis.

^c^The polymorph A percentage was estimated with the DIFFaX simulated method.

^d^The products are determined by GC-MS using a Rtx-5MS column, “1” is 1-phenylcyclopenta-necarboxylic acid, ”2” and “3” are cis-epoxide, “4” is 6-oxo-6-phenylhexanoic acid.

^e^Enantioselectivities are determined by chiral GC using a CP-Chirasil-Dex CB column, the ee value was calculated by (Selec.2 – Selec.3)/(Selec.2 + Selec.3) × 100% and the sign represented the enantiomer excess.
