# Supplementary material for: Synthesis of chiral polymorph A-enriched zeolite Beta with an extremely concentrated fluoride route
Source: Sci Rep. 2015 Jun 22;5:11521. doi: 10.1038/srep11521 (PMC4476144; doi:10.1038/srep11521)
Supplement: Supplementary Information [file srep11521-s1.pdf]

Synthesis of chiral polymorph A-enriched zeolite Beta with an extremely concentrated fluoride route

Mingquan Tong[1], Daliang Zhang[1], Weibin Fan[2], Jun Xu[3], Liangkui Zhu[1], Wen Guo[1], Wenfu Yan[1,*], Jihong Yu[1], Shilun Qiu[1], Jianguo Wang[2], Feng Deng[3], and Ruren Xu[1,]

[1] State Key Laboratory of Inorganic Synthesis and Preparative Chemistry, Department of Chemistry, Jilin University, Changchun, 130012, China

[2] State Key Laboratory of Coal Conversion, Institute of Coal Chemistry, Chinese Academy of Sciences, Taiyuan, 030001, China

[3] State Key Laboratory of Magnetic Resonance and Atomic and Molecular Physics, Wuhan Institute of Physics and Mathematics, Chinese Academy of Sciences, Wuhan, 430071, China

[*]Corresponding. yanw@jlu.edu.cn (W.Y.)

Supporting Figures

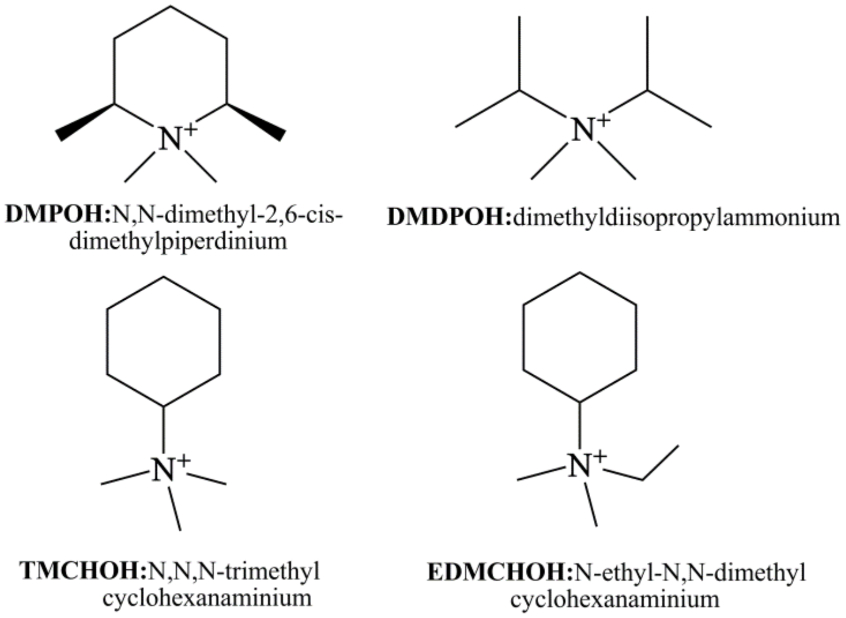

Scheme S1. The synthesized OSDAs employed in this work.

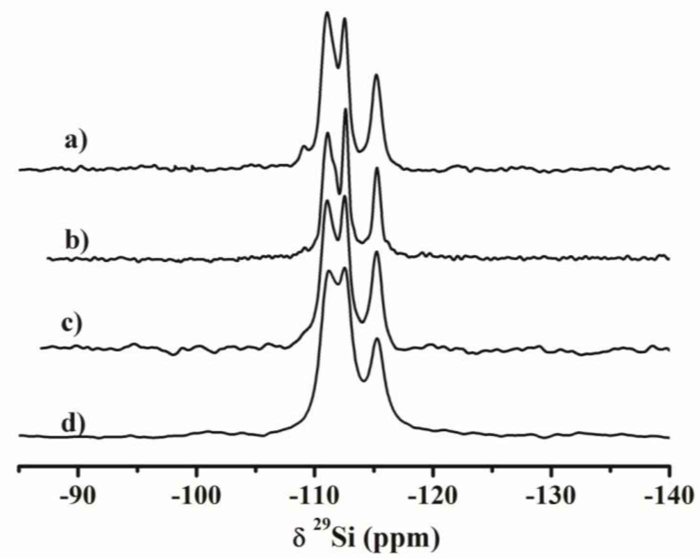

Figure S1. ^{29}Si MAS NMR spectra of the calcined normal zeolite Beta (a), Beta-TEAOH (b), Beta-DMPOH (c), and Beta-DMDPOH (d).

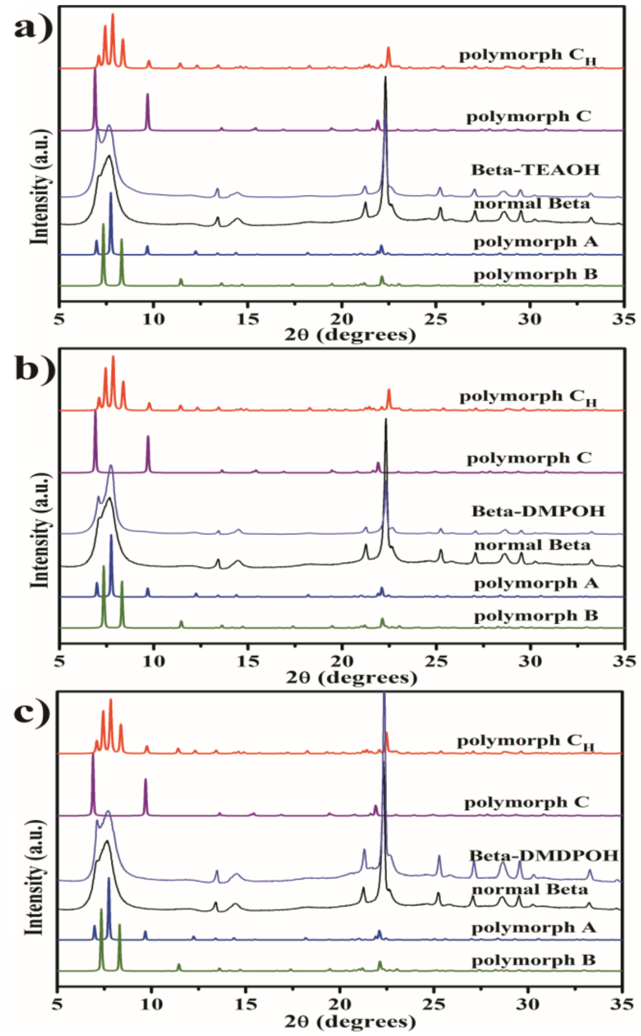

Figure S2. Comparison of the powder XRD patterns of the normal and polymorph A-enriched zeolite Beta and the simulated ones of the pure polymorph A, B,C, and C_H: (a) Beta-TEAOH, (b) Beta-DMPOH, and (c) Beta-DMDPOH.

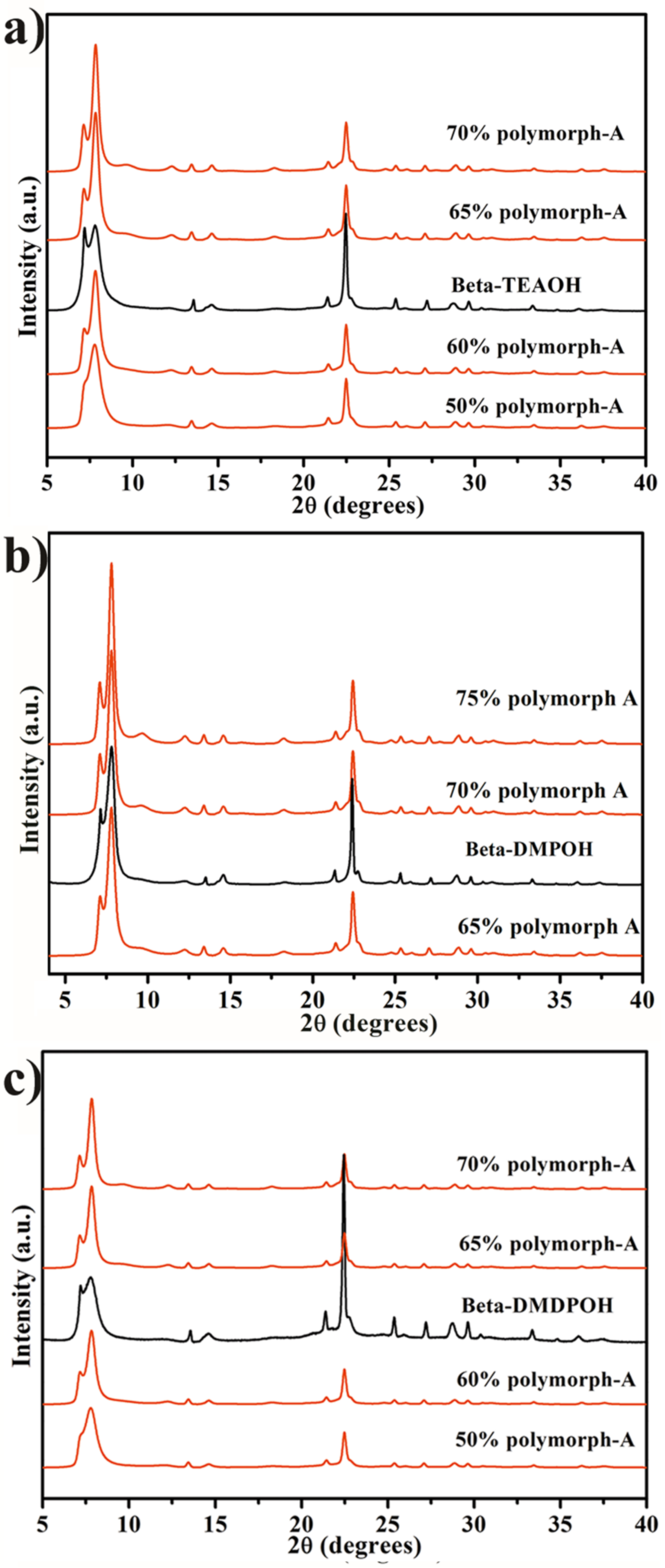

Figure S3. The experimental and simulated powder XRD patterns of the zeolite Beta with different ratios of A/B: (a) Beta-TEAOH, (b) Beta-DMPOH, (c) Beta-DMDPOH.

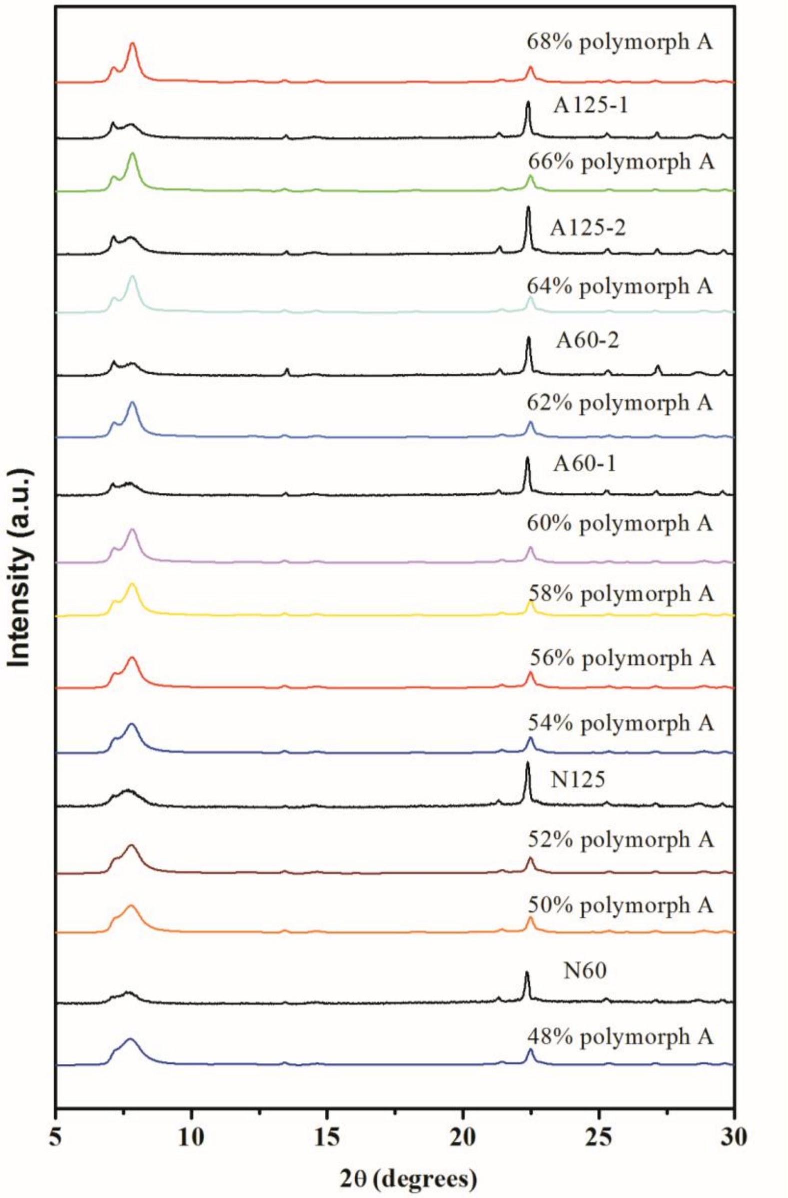

Figure S4. Experimental and simulated powder XRD patterns of Ti-Beta with different ratios of polymorph A to B

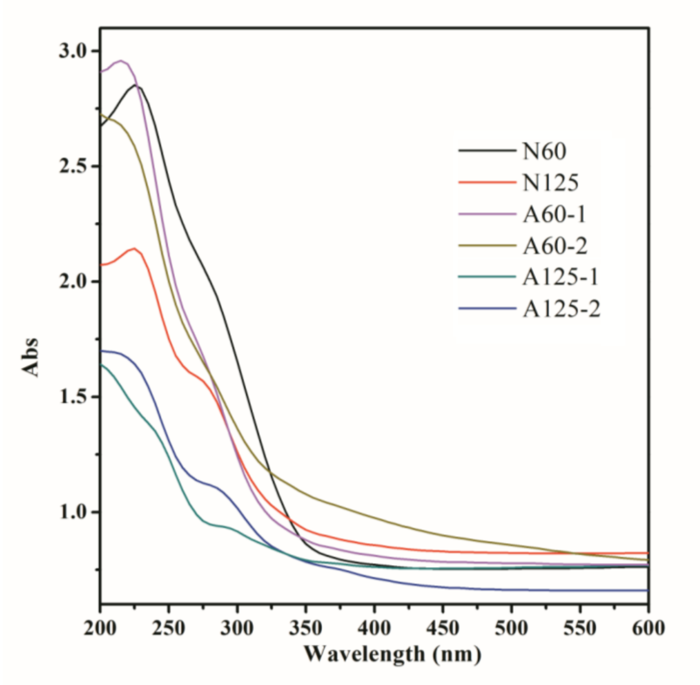

Figure S5. DR UV-vis spectra of the Ti-Beta with different ratios of polymorph A to B

Figure S5 shows that all the samples contain tetrahedral and octahedral Ti species. Despite that the amount of tetrahedral Ti species increased with increasing Ti amount in the synthesis gel, it depends on the synthesis method. Compared to N125, much smaller amounts of tetrahedral Ti species were present in the A125-1 and A125-2 maybe due to the presence of significantly less amounts of water in the synthesis gel, and hence, the different crystallization mechanism.

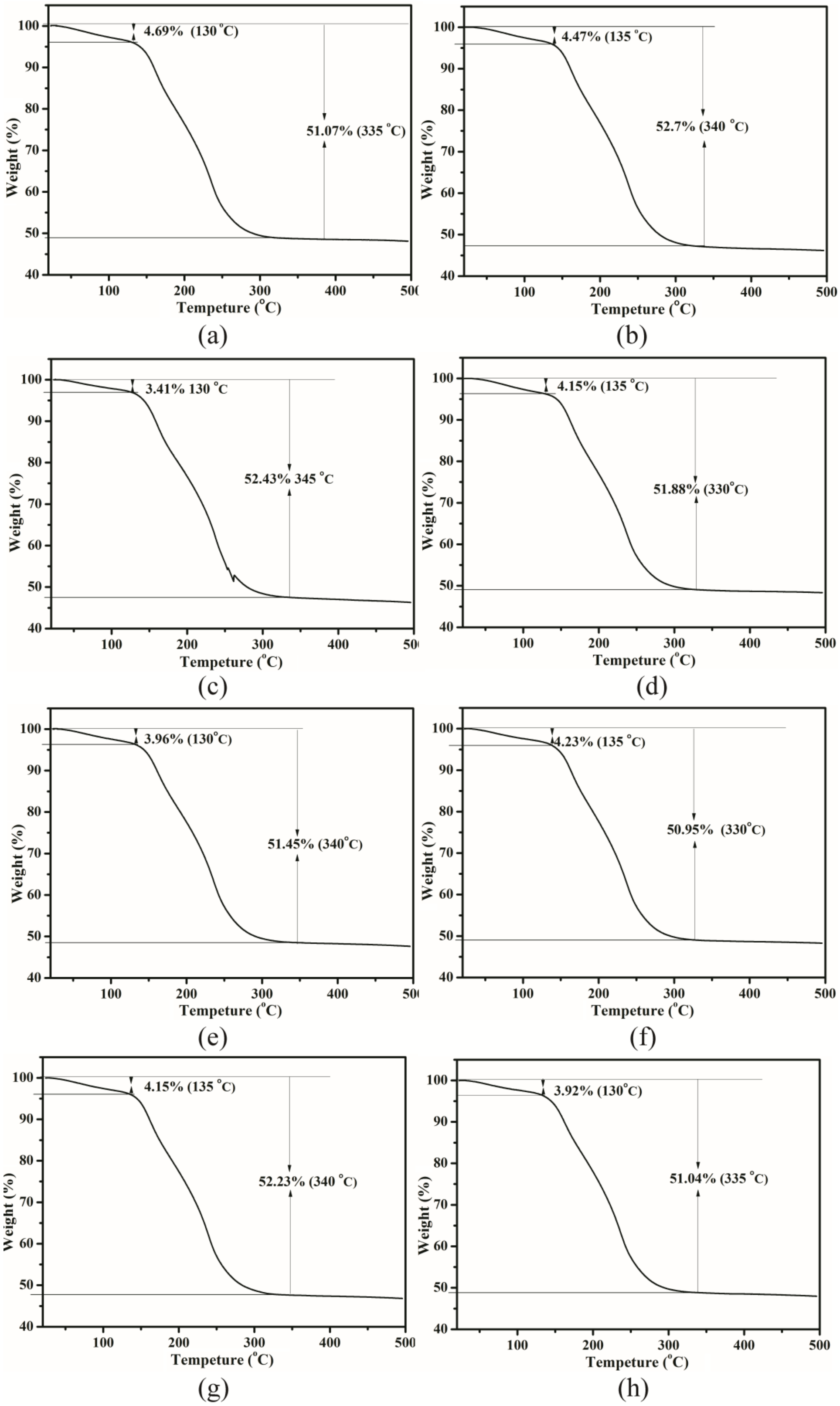

Figure S6. TGA curves of the initial mixtures in the reproduction of polymorph A-enriched zeolite beta: (a) batch 1; (b) batch 2; (c) batch 3; (d) batch 4; (e) batch 5; (f) batch 6; (g) batch 7; (h) batch 8.

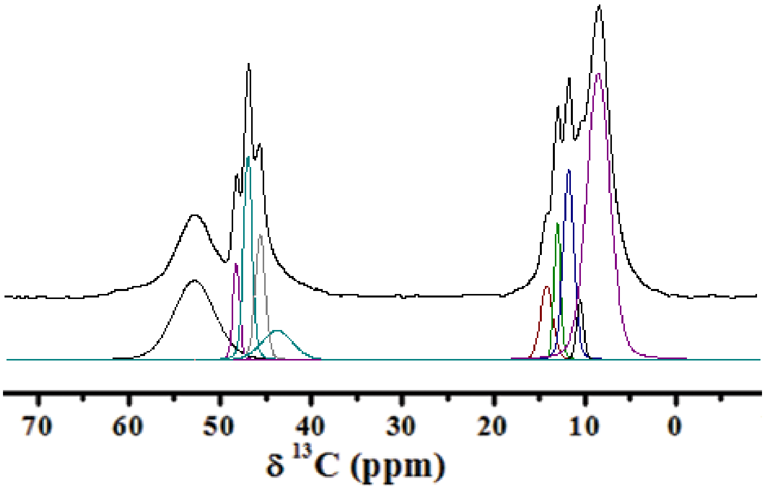

Figure S7. ^{13}C MAS NMR spectra of an initial mixture. The high field signals at 8.7~14.3 ppm are ascribed to the methyl group of TEAOH and triethylamine derived from decomposition of TEAOH in different chemical environment, while the methylene group of these alkylamine locates at 43.8~52.9 ppm. The concentration of residual TEAOH and the formed TEA is determined by ^{13}C spin counting of the methylene signal at 52.9 ppm and 45.72~48.34 ppm, respectively.

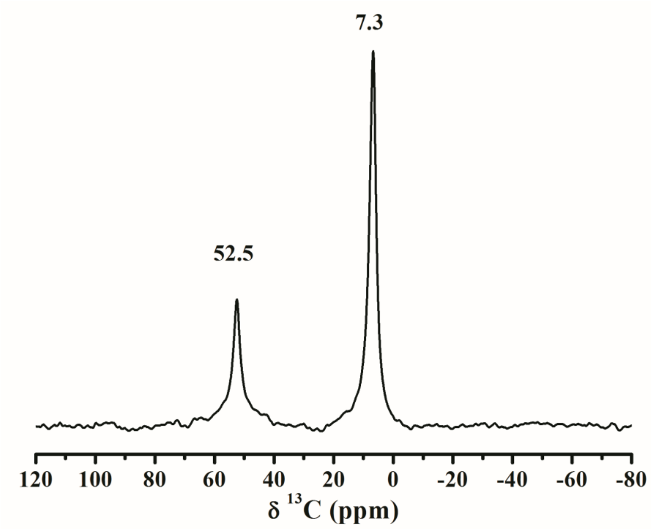

Figure S8. ^{13}C MAS NMR spectrum of beta-TEAOH

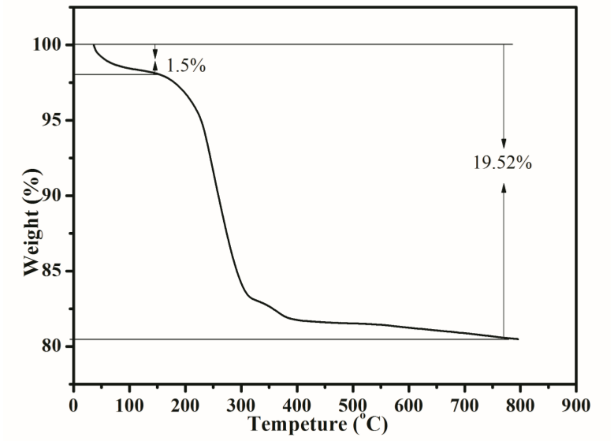

Figure S9. TGA curve of beta-TEAOH

Table S1. Catalytic results of polymorph A-enriched Ti-beta-TEAOH and normal Ti-beta for asymmetric epoxidation of β-methylstyrene

Catalyst No.[a]	Si/Ti[b]	Poly A (%)[c]	Conv.	TON	Selec.		ee_{epo}%[d]
					R,R_{epo}	S,S_{epo}	
A60-1	58	65	11.7	4.00	15.06	14.44	2.10
A60-2	--	63	12.1		14.32	15.75	-4.76
A60-3	--	66	12.3		13.98	14.27	-1.03
A125-1	126	68	6.4	4.70	15.63	17.51	-5.67
A125-2	--	67	8.5		16.22	15.49	2.30
N125-1	94	53	7.0	3.85	12.00	12.03	-0.12
N60-1	42	51	17.6	4.40	12.97	13.10	-0.50

[a] "A" and "N" represent polymorph A-enriched and normal zeolite beta, respectively; "125" and "60" represent the Si/Ti ratio in the initial mixture. [b] The Si/Ti ratios of the bulk samples were determined by ICP analysis. [c] The polymorph A percentage was estimated with the DIFFaX simulated method. [d] Enantioselectivities for the SS and RR epoxides are determined by chiral GC equipped with a CP-Chirasil-Dex CB column, the ee value was calculated by (Selec.$_{R,R}$ – Selec.$_{S,S}$)/(Selec.$_{R,R}$ + Selec.$_{S,S}$) ×100% and the sign represents the enantiomer excess.

Table S2. The summary of the results in the TGA analyses of the eight initial mixtures. The average weight losses during the first and second step are 4.12% and 47.70%, respectively.

Sample No.	Total weight loss (%)	Weight loss duringthe first step		Weight loss during the second step	
		Temperature (°C)	Weight loss (%)	Temperature (°C)	Weight loss (%)
1	51.07	130	4.69	335	46.38
2	52.70	135	4.47	340	48.23
3	52.43	130	3.41	345	49.02
4	51.88	135	4.15	330	47.73
5	51.45	130	3.96	340	47.49
6	50.95	135	4.23	330	46.72
7	52.23	135	4.15	340	48.08
8	51.04	130	3.92	335	47.12
